# Detection of *Cryptosporidium* spp. and *Giardia duodenalis* in small wild mammals in northeastern Brazil

**DOI:** 10.1371/journal.pone.0256199

**Published:** 2021-08-16

**Authors:** Hllytchaikra Ferraz Fehlberg, Cássia Matos Ribeiro, Pedro de Alcântara Brito Junior, Bruno César Miranda Oliveira, Camila Albano dos Santos, Martín Roberto del Valle Alvarez, Tatiane Vitor Harvey, George Rêgo Albuquerque

**Affiliations:** 1 Department of Agricultural and Environmental Sciences, Santa Cruz State University—UESC, Ilhéus, BA, Brazil; 2 Department of Support, Production and Animal Health, Universidade Estadual Paulista—UNESP, Araçatuba, SP, Brazi; 3 Department of Biological Sciences, State University of Santa Cruz—UESC, Ilhéus, BA, Brazi; University of Bari, ITALY

## Abstract

This study investigated the occurrence of *Giardia duodenalis* and *Cryptosporidium* spp. in rodents and marsupials from the Atlantic Forest in southern Bahia, northeastern Brazil. Two hundred and four fecal samples were collected from different forest areas in the municipalities of Ilhéus, Una, Belmonte, and Mascote. Identifications were performed using PCR and nested PCR followed by sequencing of the *gdh* and *tpi* genes for *G*. *duodenalis*, and the *gp60* and *Hsp-70* genes for *Cryptosporidium*. The total frequency of positive PCR samples for both *G*. *duodenalis* and *Cryptosporidium* spp. was 5.4% (11/204). *Giardia duodenalis* occurred in 2.94% (4/136) of rodents and 2.94% (2/68) of marsupials. The prevalence of *Cryptosporidium* in rodents and marsupials was 1.47% (2/136) and 4.41% (3/68), respectively. In the areas sampled, the frequency of parasitism was 50% (7/14), while the Mascote region alone had no parasitized animals. The *G*. *duodenalis* subgenotype AI was identified in the rodent species *Hylaeamys laticeps*, *Oecomys catherinae*, *Oligoryzomys nigripes* and *Akodon cursor*, and in the marsupials *Gracilinanus agilis* and *Monodelphis americana*. In the rodents *Rhipidomys mastacalis*, *H*. *laticeps* and in the marsupial *Marmosa murina* the protozoa *Cryptosporidium fayeri*, *Cryptosporidium parvum* and *Cryptosporidium ubiquitum* with subtypes IIa and IVg by the gp60 gene were found. In conclusion, this study provides the genetic characterization of *Giardia* and *Cryptosporidium* species and genotypes in rodents and marsupials. And, these findings reinforce that the rodent and marsupial species mentioned above play a role as new hosts for *Giardia* and *Cryptosporidium*.

## Introduction

Small mammals such as rodents (Rodentia, Cricetidae) and marsupials (Mammalia, Didelphimorphia) transmit pathogens to humans and domestic animals; however, the consequent risk to public health is poorly understood [1,2]. Environmental disruption due to human activity influences the occurrence and spread of zoonotic and parasitic diseases (e.g., giardiasis and cryptosporidiosis) in these animals, affecting the wildlife species balance [3].

*Giardia* Kunstler, 1882 and *Cryptosporidium* Tizzer, 1907 are protozoa known worldwide for causing severe gastroenteric disease in humans, as well as domestic and wild animals [2,4,5]. These protozoa cause infections from cysts or oocysts found in environmental and water contaminations [4,6].

The role of wild animals in human giardiasis and cryptosporidiosis epidemiology is uncertain. However, molecular studies have allowed the identification of several species of *Giardia* and *Cryptosporidium* in wild animals [6,7–9].

Molecular techniques have successfully determined and supported the understanding of epidemiological processes [9] by using several genes to identify distinct species of *Giardia* and *Cryptosporidium*. Additionally, they reveal genotypes and subgenotypes, of which some are specific to humans and others to animals [6].

To determine Cryptosporidium spp. genotypes and subgenotypes, coding genes stand out as small subunit 18S ribosomal rRNA (SSu-rRNA) [10]. Both gp60 and Hsp-70 demonstrate a high polymorphism in different species [11,12]. In addition, wall-protein coding genes (*COWPs*), actin, acetyl-CoA synthetase, and internal space transcribed from rDNA (*rDNA ITS 1*) are also used [13,14].

To detect the genotype and subgenotype of the *Giardia duodenalis* species, genes of *SSu-rRNA* [15,16], glutamate dehydrogenase (*gdh*), triose-phosphate isomerase (*tpi*), and beta-giardin (*bg*) coding genes are used [16–18].

Molecular studies to detect *Giardia* and *Cryptosporidium* in wildlife reported the presence of these protozoa in different species of small mammals. However, in northeastern Brazil, no studies have employed molecular genotyping to identify *G*. *duodenalis* and *Cryptosporidium* spp. Thus, the objective of this study was to identify, through a molecular technique at the level of genotypes and subgenotypes, *G*. *duodenalis* and *Cryptosporidium* spp. in fecal samples of rodents and marsupials captured in agroforestry areas (*Cabruca*) and the Atlantic Forest in southern Bahia, northeastern Brazil.

## Material and methods

### Collection area

**Fig 1:**
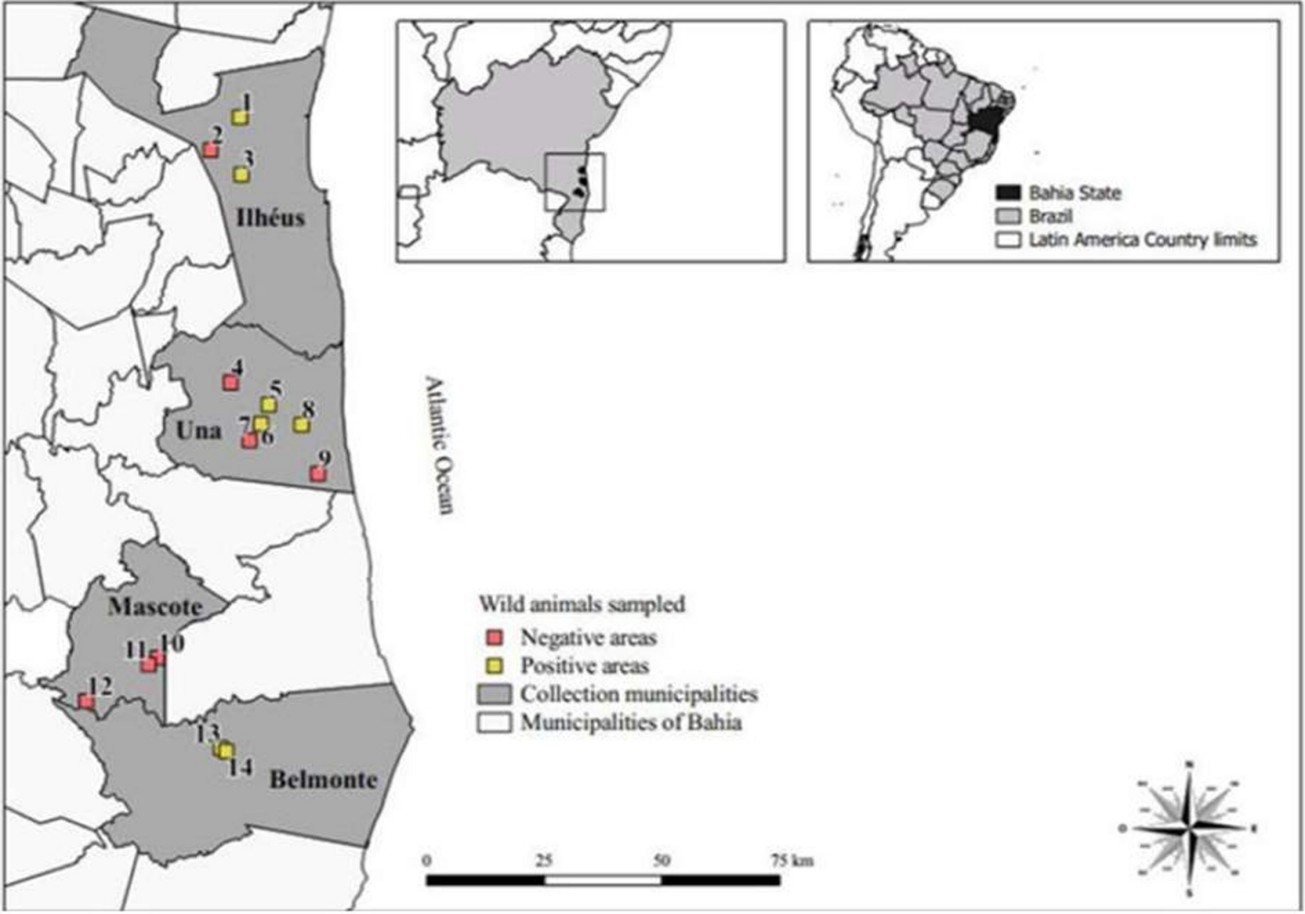
Map depicting the capture and collection areas, of fecal samples from rodents and marsupials in southern Bahia, northeastern Brazil. Geographic coordinates of the collection points. ***01*:** *14°38’15*.*8”S39°12’02*.*3”W;* ***02*:** *14°42’11*.*2”S 39°15’34*.*8”W;* ***03*:** *14°45’04*.*0”S 39°11’51*.*2”W;* ***04*:** *15°09’57*.*8”S 39°13’10*.*1”W;* ***05*:** *15°12’35*.*9”S 39°08’37*.*4”W;* ***06*:** *15°14’53*.*1”S 39°09’34*.*3”W;* ***07*:** *15°16’54*.*5”S 39°10’54*.*2”W;* ***08*:** *15°14’59*.*0”S 39°04’41*.*0”W;* ***09*:** *15°20’53*.*0”S 39°02’43*.*5”W;* ***10*:** *15°42’53*.*6”S 39°21’52*.*6”W;* ***11*:** *15°43’40*.*9”S 39°22’56*.*7”W;* ***12*:** *15°48’01*.*9”S 39°30’23*.*8”W;* ***13*:** *15°53’40*.*4”S 39°14’19*.*2”W;* ***14*:** *15°54’03*.*0”S 39°13’40*.*4”W*.

Within the study area, 14 forest areas, distributed in four municipalities in the southern region of the State of Bahia, were sampled. These included three cocoa agroforestry areas located in the rural area of Ilhéus (areas 1–3), and 11 forest areas located in the municipalities of Una, Mascote and Belmonte (areas 4–14) (Fig 1). The study region is characterized by a hot and humid tropical climate, with an average relative humidity of 89–90% and an average temperature of 24–25°C, predominantly covered by tropical forest vegetation and an agroforestry system, which preserves native forest [19]. In the region, it rains 150 days a year on average, with precipitation reaching 2,000 mm/year. The dry seasons are not well defined; occasionally, one to three months receive less than 100 mm of rain [20]. Elevation of the sampled areas ranged from 42–100 m above sea level and were georeferenced with a Global Positioning System (GPS).

### Capturing animals and obtaining biological material

The capture period ranged from June 2015 to December 2016. The animals were captured using Sherman (23 × 8 × 9 cm), Tomahawk (50 × 17 × 17 cm), and pitfall traps. Each area was divided into three plots, with for a total of 24 traps per plot and 72 traps per area. The study was approved by the Biodiversity Authorization and Information System (SISBIO) under number 17131–4 from the Brazilian Institute for the Environment and Renewable Natural Resources (IBAMA) and by the Council for the Ethical Use of Animals of the State University of Santa Cruz (CEUA-UESC; Case No. 003/2013).

After identification of the species, fecal samples were collected with subsequent release of the animals at the places of origin (Table 1). Fecal samples were stored in 1.5 mL microtubes, kept refrigerated and delivered to Laboratory of Veterinary Parasitology of the State University of Santa Cruz (LAPVET-UESC), weighed, and standardized between 180 and 200 mg.

**Table 1:** Species of marsupials and wild rodents captured in the Atlantic Forest and *Cabruca* areas in southern Bahia, northeastern Brazil, and positivity of infected animals.

	**Area**	N*/Positives	**Molecular diagnosis (*Nested*/PCR)**	
**ORDER DIDELPHIMORPHIA**				
**Family Didelphidae**			***Cryptosporidium***	***Giardia***
*Marmosa murina* (Linnaeus, 1758)				
	3;4;6;7;8;9;10;11;12;13;14	26/3	3	0
*Marmosa incanus* (Lund, 1840)	11; 13	7/0	0	0
*Marmosa demerarai* (Thomas, 1905)	4;7;8	9/0	0	0
*Monodelphis americana* (Müller, 1776)	3;4;14	8/1	0	1
*Gracilinanus agilis* (Burmeister, 1854)	12;14	10/1	0	1
*Didelphis aurita* (Wied-Neuwied, 1826)	7;8	8/0	0	0
**TOTAL**		**68/5**	**3**	**2**
**ORDER RODENTIA**				
**Family Cricetidae**				
*Hylaeamys laticeps* (Lund, 1840)				
	1;2;3;4;5;8	81/2	1	1
*Akodon cursor* (Winge, 1887)	1;2;3;11;14	13/1	0	1
*Rhipidomys mastacalis* (Lund, 1840)	1;2;3;5;8;12;13	11/1	1	0
*Thaptomys nigrita* (Lichtenstein, 1829)	1;5;8;13;14	9/0	0	0
*Oecomys catherinae* (Thomas, 1909)	5;7;8;13	5/1	0	1
*Calomys expulsus* (Lund, 1841)	12	2/0	0	0
*Cerradomys subflavus* (Percequillo et al., 2008)	1;2;11	4/0	0	0
*Oligoryzomys nigripes* (Olfers, 1818)	1;2;5;7;8;12	7/1	0	1
*Euryoryzomys russatus* (Wagner, 1848)	1;3;13	4/0	0	0
**TOTAL**		**136/6**	**2**	**4**
**GRAND TOTAL**		**204/11**	**5**	**6**

### DNA extraction and molecular characterization

The fecal samples were washed with sterile PBS (pH 7.2) and subjected to genomic DNA extraction using the QIAamp DNA Stool Mini kit^*®*^ (Qiagen), according to manufacturer’s instructions. After adding the lysis buffer, the samples were subjected to five cycles of heating (96°C) and freezing (-196°C), with 3 minutes of heating and 5 minutes of freezing, then homogenized in a vortex for 5 minutes with 0.2 g of glass beads (0.5 mm), following the kit’s guidelines thereafter. The amount of extracted genomic DNA was established using a NanoDrop 2000 (Thermo Scientific, USA), stored in boxes, and placed in a freezer at -20°C.

To detect the presence of *G*. *duodenalis* and *Cryptosporidium* spp., each isolated DNA sample was subjected to nested PCR. For the amplification of *Giardia* fragments, *gdh* [16] and *tpi* coding genes [17] were used. *Cryptosporidium* fragments were amplified using *gp60* [12] and *Hsp-70* [11] genes (Table 1).

The tests were carried out in a Proflex PCR system thermocycler (Applied Biosystems) using the Platinum Taq DNA polymerase kit (Invitrogen) for the mix. Positive fecal samples from *Giardia* cysts and isolates from the Veterinary Parasitology Laboratory at UESC were used as positive controls. *Cryptosporidium* (isolates 13F and 13C) from the Laboratory of Clinical Analysis (LAC) of the State University of Feira de Santana, Bahia [21] and ultrapure water were used as negative controls. The PCR products were subjected to 1% agarose gel electrophoresis, developed with SYBR^®^ Safe, purified using the PureLink PCR Purification kit (Invitrogen), and sent for sequencing.

Sequencing was performed using capillary electrophoresis (modified Sanger sequencing) on the ABI 3500XL Genetic Analyzer platform (Applied Biosystems) in both directions. Chromatogram analysis was performed using the FinchTV 1.4.0 software. Amplicons were Sanger-sequenced in both directions. DNA sequences were deposited in GenBank under accession numbers MW202351, MW202352, MW202353, MW202354, MW202355, MW202356, MW202357, MW202358, MW202359, MW202360, MW202361, MW202362, MW202363, MW202364, MW202365, MW202366 and MW202367.

### Statistical analysis

To verify the association between the positivity of the samples with the catch area (agroforestry and forest areas), statistical analysis was performed using Fisher’s exact test with 95% confidence intervals using the Epi Info ™ 7.2.0.1 software.

## Results

Out of 204 fecal samples collected, 5.4% (11/204) tested positive (Table 1). The occurrence of *G*. *duodenalis* was 2.94% (6/204) for rodents 2.94% (4/136), and marsupials 2.94% (2/68) (Table 2). For *Cryptosporidium*, the combined positivity was 2.45% (5/204), with 1.47% (2/136) and 4.41% (3/68) for rodents and marsupials, respectively (Table 3). In the collection areas, the frequency of parasitism was 50% (7/14) and there were no parasitized animals in the municipality of Mascote (Fig 1). The agroforestry areas had the highest frequency of infected animals, although the differences between the positivity in capture areas were not statistically significant (p> 0.05).

**Table 2:** Species of *Giardia* per parasitized host caught in forest and *Cabruca* areas in southern Bahia, northeastern Brazil.

Hosts	PCR marker			Subgenotypes
Species	Order	*TPI*	*GDH*	
*Gracilinanus agilis*	Didelmorphia	Gd	Gd	AI*
*Monodelphis americana*	Didelmorphia	Gd	Gd	AI
*Oecomys catherinae*	Rodentia	Gd	Gd	AI
*Oligoryzomys nigripes*	Rodentia	Gd	Gd	AI
*Hylaeamys laticeps*	Rodentia	Gd	Gd	AI
*Akodon cursor*	Rodentia	Gd	Gd	AI

Abbreviations: Gd: *Giardia duodenalis*.

*Subgenotype.

**Table 3:** Species of *Cryptosporidium* per parasitized host caught in forest and *Cabruca* area in southern Bahia, northeastern Brazil.

Hosts		PCR marker		*Gp60* subgenotype family
Species	Order	*HSP-70*	*Gp60*	
*Marmosa murina*	Didelmorphia	Cp	Cp	IIa*
*M*. *murina*	Didelmorphia	Cr	Cf	IVg*
*M*. *murina*	Didelmorphia	Cr	Cp	IIa
*Rhipidomis mastacalis*	Rodentia	Cp	Cp	IIa
*Hylaeamys laticeps*	Rodentia	Cu		

Abbreviations: Cp: *Cryptosporidium parvum*; Cf: *Cryptosporidium fayeri*; Cr: *Cryptosporidium* sp.; Cu: *Cryptosporidium ubiquitum*.

* Subgenotype.

The analysis of the *tpi* and *gdh* gene sequences demonstrated 100% genetic similarity with the *G*. *duodenalis* species of the subgenotype AI (Table 2). The genetic analysis of *Cryptosporidium* identified *C*. *parvum*, *C*. *ubiquitum*, and *C*. *fayeri*, and subtypes that belong to the IIa and IVg allelic families. No subtype found for *C*. *ubiquitum* (Table 3).

## Discussion

The present study investigated, for the first time, the presence of the protozoa *Giardia* and *Cryptosporidium* in rodents and marsupials captured in the northeast region of Brazil. The southern region of Bahia includes an extensive area of the Atlantic Forest with a richness of fauna and flora species, being an important area for the conservation of global biodiversity [20]. In addition to having areas of cocoa agroforestry, providing shade for planting and preserving native forests [22].

*Giardia duodenalis* infection has been described in wild animals, such as rodents and marsupials, with a prevalence ranging from 2% to 12% [3,23–27]. This defines a low prevalence in forest areas, compared to that in urban areas with rodents having a higher prevalence ranging from 24.4% to 64.3% [2,23,28]. In the present study, the frequency of positive animals was 5.4%, and such low positivity may be related to the sampling site, which has rich and abundant flora, low anthropization, and the presence of some arboreal animal species, such as *G*. *agilis* and *O*. *catherinae*, which have herbivorous and insectivorous diet, respectively [26,29,30] reducing contact with the pathogen.

The subgenotype AI found in this study is commonly found in humans [31], which characterizes these animals as participants in the epidemiology of human *Giardia* infection [25]. Vermeulen et al. [25], Caccio and Ryan [32], Karim et al. [33], and Garcia et al. [34] identified the same subgenotype in the *gdh* and *tpi* genes in animals. Marsupials and rodents, especially those which are terrestrial, such as the marsupials *M*. *murina* and *M*. *americana*, and the rodents *O*. *nigripes*, *H*. *laticeps*, *A*. *cursor*, and *R*. *mastacalis*, become infected through contaminated water, food, and fomites, thus playing an important role in the evolution of this protozoan [29]. Additionally, this brings the parasite into contact with humans, presenting a risk to public health [31,35].

The *gdh* and *tpi* genes demonstrated good sensitivity, allowing the generated sequences to identify the *G*. *duodenalis* species and the subgenotype AI in the six isolates. Because it has conserved regions, characterization of these genes can identify all genotypes and subgenotypes of *G*. *duodenalis* [36–38].

The *Cryptosporidium* frequency was 1.47% and 4.41% in rodents and marsupials, respectively, similar to that described by Santos [24]. The literature describes this protozoan infecting a variety of small mammal species [3,24,39–44]. Studies in urban areas also show a greater degree of parasitism of this protozoan in synanthropic rodents [2,28,41,42]. The presence of this protozoan may be associated with anthropic action and the presence of domestic animals provides an interaction between humans and wild fauna, favoring its dissemination [45].

*Cryptosporidium parvum* is responsible for the majority of human enteric infections worldwide [44]. The subgenotype IIa obtained in this study is frequently found in humans and animals [43,44,46–48]. *Cryptosporidium fayeri* is common in marsupial species [40,44,49,50] despite has also been identified in humans [44,51,52]. Its pathogenicity is unknown, but it often causes asymptomatic infections in marsupials [40]. The subgenotype IVg has been identified in marsupials (*Macropus giganteus*) [44].

*Cryptosporidium ubiquitum* was found in *Hylaeamys laticeps*, the first finding in wild rodents captured in Brazil. This species has low specificity and is commonly reported in animals, including rodents, marsupials, and other host species [35,41,43,53,54]. Cases in humans have shown that [55,56] the most common route of *C*. *ubiquitum* transmission is through water [56].

The two genes assessed, *gp60* and *Hsp-70*, have satisfactory sensitivity and can be used in studies to identify *Cryptosporidium* and verify its genetic diversity [45,53,57,58]. Using more than one gene provides a more detailed understanding of the protozoan’s genetic variability and abiotic factors in the study population [59].

In this study, the occurrence of protozoa in small mammals was similar in the Atlantic Forest (Una and Belmonte) and agroforestry (Ilhéus) environments. The difference in the number of positive animals between capture areas was not statistically significant, demonstrating that agroforestry areas maintain low contamination due to the continued diversity of fauna and flora, despite greater anthropic action and transit of domestic animals that threaten the diversity of wild animals [60].

The close human relationship with wildlife as a result of disorderly urban occupation, illegal trade in wild animals, or the maintenance of these animals as pets, are some of the factors that enhance the transmission of zoonotic diseases between species, thus threatening both conservation of biodiversity, and public health [61,62]. Thus, surveillance and monitoring of wildlife pathogens is necessary for the detection, mitigation and prevention of diseases with zoonotic potential.

## Conclusion

Results herein obtained pioneer Giardia and Cryptosporidium identification in rodents and marsupials from southern Bahia, northeastern Brazil, showing the present technique as sensitive enough to identify the subgenotypes of Giardia and Cryptosporidium through the gdh and tpi, and Hsp-70 and gp60 genes, respectively.
